# Epidemiological investigation of a temporal increase in atonic postpartum haemorrhage: a population-based retrospective cohort study

**DOI:** 10.1111/1471-0528.12149

**Published:** 2013-03-06

**Authors:** A Mehrabadi, JA Hutcheon, L Lee, MS Kramer, RM Liston, KS Joseph

**Affiliations:** a Department of Obstetrics and Gynaecology, University of British Columbia and the Children's and Women's Health Centre of British ColumbiaVancouver, BC, Canada; b School of Population and Public Health, University of British ColumbiaVancouver, BC, Canada; c Perinatal Services BCVancouver, BC, Canada; d Departments of Pediatrics, Epidemiology, Biostatistics and Occupational Health, McGill UniversityMontreal, QC, Canada

**Keywords:** Atonic postpartum haemorrhage, postpartum haemorrhage, severe maternal morbidity, uterine atony

## Abstract

**Objective:**

Increases in atonic postpartum haemorrhage (PPH) have been reported from several countries in recent years. We attempted to determine the potential cause of the increase in atonic and severe atonic PPH.

**Design:**

Population-based retrospective cohort study.

**Setting:**

British Columbia, Canada, 2001–2009.

**Population:**

All women with live births or stillbirths.

**Methods:**

Detailed clinical information was obtained for 371 193 women from the British Columbia Perinatal Data Registry. Outcomes of interest were atonic PPH and severe atonic PPH (atonic PPH with blood transfusion ≥1 unit; atonic PPH with blood transfusion ≥3 units or procedures to control bleeding), whereas determinants studied included maternal characteristics (e.g. age, parity, and body mass index) and obstetrics practice factors (e.g. labour induction, augmentation, and caesarean delivery). Year-specific unadjusted and adjusted odds ratios for the outcomes were compared using logistic regression.

**Main outcome measures:**

Atonic PPH and severe atonic PPH.

**Results:**

Atonic PPH increased from 4.8% in 2001 to 6.3% in 2009, atonic PPH with blood transfusion ≥1 unit increased from 16.6 in 2001 to 25.5 per 10 000 deliveries in 2009, and atonic PPH with blood transfusion ≥3 units or procedures to control bleeding increased from 11.9 to 17.6 per 10 000 deliveries. The crude 34% (95% CI 26–42%) increase in atonic PPH between 2001 and 2009 remained unchanged (42% increase, 95% CI 34–51%) after adjustment for determinants of PPH. Similarly, adjustment did not explain the increase in severe atonic PPH.

**Conclusions:**

Changes in maternal characteristics and obstetric practice do not explain the recent increase in atonic and severe atonic PPH.

## Introduction

Increases in postpartum haemorrhage (PPH) and severe PPH have been reported in Australia, Canada, Ireland, Scotland, Norway, Sweden, and the USA over the last two decades.^1^–^9^ The increases were driven by rising rates of atonic and other early PPH following the delivery of the placenta.^2^,^3^,^5^ Although maternal deaths are rare in developed countries,^10^ the observed increase in PPH is concerning because PPH is an important cause of maternal death and the most common severe maternal morbidity.^11^ Isolating the cause of the rise in atonic PPH is key to identifying prevention strategies.

Recent studies have examined various potential causes for the temporal increase in PPH, including changes in maternal and pregnancy characteristics, such as increases in older maternal age, obesity, and multiple pregnancies, and changes in obstetric practice, including increases in labour induction and caesarean delivery.^1^,^5^,^6^,^9^,^12^,^13^ However, the cause(s) for the temporal increase in atonic PPH remain unclear, and there is some suspicion that changes in the diagnosis of postpartum haemorrhage may have been partly responsible for the rising rates.

We carried out a population-based study to identify the potential cause of the temporal increase in atonic PPH using data from British Columbia, Canada, where atonic PPH has increased in recent years.^14^ Our choice of this sub-national population was motivated by the high-quality and detailed nature of the data available for analysis. This enabled an examination of the impact of changes in several maternal characteristics (such as pre-pregnancy weight) and obstetric practice (such as the role of labour induction and augmentation) not previously examined in a population-based study. Finally, we examined changes in both atonic PPH and severe atonic PPH (measured using two different markers of severity) in order to ascertain if the over-diagnosis of borderline atonic PPH was potentially responsible for the increases in PPH rates.

## Methods

The study population included all women resident in British Columbia who delivered between 1 April 2001 and 31 March 2010 (hereafter referred to as 2001–2009). Data were obtained from the British Columbia Perinatal Data Registry, a population-based registry that contains information on approximately 99% of births in the province, including home births attended by registered midwives. Each woman has a standard form that is filled out by care providers antenatally, during labour and delivery, and postpartum, and women's medical charts are then abstracted by trained hospital personnel or database registry staff. Data quality checks and the continued surveillance and research use of the database ensure the accuracy of the information stored.

The outcomes of interest were any atonic PPH and severe atonic PPH. PPH was defined as an estimated blood loss of ≥500 ml after vaginal delivery or >1000 ml after caesarean delivery, or as otherwise diagnosed and documented by the healthcare provider. Estimated postpartum blood loss is routinely collected by healthcare providers as a range of <500, 500–1000 or >1000 ml, and then used by the data abstracters in conjunction with other chart information to code PPH. The ninth revision of the International Classification of Diseases (ICD-9) code 666.1 and the ICD-10 code O72.1 were used to identify atonic PPH (haemorrhage occurring within the first 24 hours of delivery, not including PPH caused by retained placenta or coagulation defects). Temporal trends were also examined for the other subtypes of PPH, including PPH caused by a retained placenta, secondary PPH (PPH more than 24 hours after delivery), and PPH caused by coagulation defects.

There were two binary measures of severe atonic PPH. First, atonic PPH in conjunction with blood transfusion of one or more units of whole blood or packed red blood cells following delivery. Second, a composite outcome that represented very severe atonic PPH, was defined as atonic PPH with any of the following: (i) receipt of three or more units of whole blood or packed red blood cells; (ii) emergency hysterectomy; (iii) uterine (and vaginal) packing; (iv) ligation of pelvic vessels; or (v) embolisation of pelvic vessels. The composite outcome was used to account for potential changes in obstetric practice that may have reduced the threshold for blood transfusion. Hysterectomy and procedures to control bleeding were identified using the Canadian Classification of Health Interventions codes (Table S1 lists the diagnosis and procedure codes used).

Temporal trends in the rates of atonic PPH, severe atonic PPH, and maternal, fetal, and obstetric characteristics were assessed both by contrasting the rates in 2009 versus 2001 (using rate ratios and 95% CIs) and by examining the trend across all years using a chi-square test for linear trend in proportions. All rates were expressed per 100 deliveries, except for rare outcomes such as severe PPH, which were expressed per 10 000 deliveries. Calendar time was also modelled using restricted cubic splines in order to explore a nonlinear relationship with the outcome. Covariates were modelled using continuous or categorical variables after assessing assumptions regarding linearity and assessing the ease of interpretation. In order to account for missing pre-pregnancy body mass index (BMI) data, multiple imputation was used to impute 20% missing data for height and 23% missing data for pre-pregnancy weight. Multiple imputation assumed an underlying multivariate normal model, composed of 20 imputed data sets, and included all study covariates and outcomes as model covariates.

The three outcomes (atonic PPH, atonic PPH with blood transfusion ≥1 unit, and atonic PPH with blood transfusion ≥3 units or a procedure to control bleeding) were modelled separately using logistic regression. As the definition of PPH varied by mode of delivery (≥500 ml for vaginal and >1000 ml for caesarean delivery), sensitivity analyses modelling atonic PPH separately among vaginal and caesarean deliveries were also carried out. We further carried out sensitivity analyses in which covariates were added incrementally to the model, starting with maternal pre-pregnancy factors, followed by maternal pregnancy factors, and lastly obstetric factors, namely, labour induction and labour augmentation, epidural analgesia, and mode of delivery.

The crude odds ratio and 95% CI expressing the period effect (2009 versus 2001) was compared with the adjusted odds ratio and 95% CI to determine if the adjustment for changes in maternal characteristics and obstetric practice explained the temporal trend in atonic and severe atonic PPH. We anticipated that the crude odds ratios expressing the temporal increase in atonic PPH and severe atonic PPH would be attenuated (i.e. closer to 1) after adjustment for risk factors that were responsible for the rise in atonic PPH and severe atonic PPH. Generalised estimating equations, with an assumed unstructured correlation structure, were used to account for repeat pregnancies to the same woman during the study period. Statistical significance was assessed based on two-sided *P* values and *P* < 0.05 was considered to be statistically significant. Analyses were carried out using sas 9.2 and stata SE 11. The study received ethics approval from the University of British Columbia Children's & Women's Health Centre Research Ethics Board (ref. no. H11–00307).

## Results

**Figure 1:**
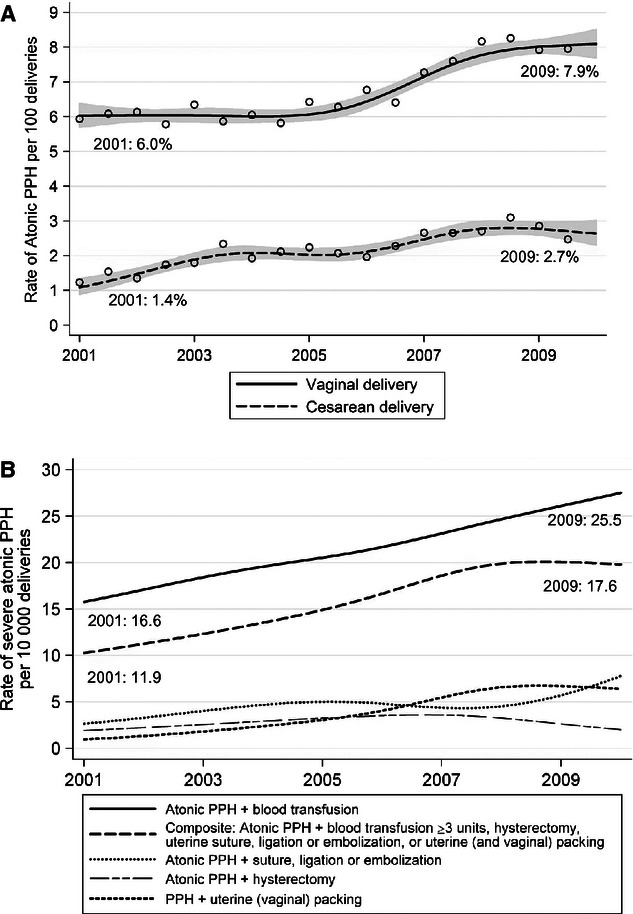
Temporal trends in atonic postpartum haemorrhage (PPH) by vaginal versus caesarean delivery, smoothed rates with 95% confidence intervals overlaid on observed semi-annual rates (A), and severe atonic PPH as measured by PPH in conjunction with various markers of severity, smoothed rates (B).

There were 372 259 deliveries to residents of British Columbia between 2001 and 2009. We restricted our analyses to 371 193 deliveries that had complete information for gestational age and birthweight (0.3% missing). The rate of atonic PPH increased from 4.8% in 2001 to 6.3% in 2009 (34% increase in unadjusted odds, 95% CI 26–42%). Among vaginal deliveries, atonic PPH increased by 35% (95% CI 27–44%) from 6.0% in 2001 to 7.9% in 2009, whereas atonic PPH among caesarean deliveries increased by 95% (95% CI 61–137%) from 1.4% to 2.7% (Figure 1A). The rate of atonic PPH with blood transfusion ≥1 unit increased by 51% (95% CI 11–104%) from 16.6 to 25.5 per 10 000 deliveries from 2001 to 2009, whereas the composite outcome for severe atonic PPH with blood transfusion ≥3 units or procedures to control bleeding increased by 47% (95% CI 2–112%) from 11.9 in 2001 to 17.6 per 10 000 deliveries in 2009. Atonic PPH with blood transfusion ≥1 unit increased steadily between 2001 and 2009, whereas uterine suturing, ligation, or embolisation increased most markedly between 2005 and 2008. Hysterectomy for atonic PPH increased until 2006, and then decreased (Figure 1B). No significant changes were observed for other subtypes of PPH, including PPH caused by retained placenta, secondary PPH, and PPH caused by coagulation defects (all *P* values for linear trend in proportions >0.05). Nor were marked nonlinear trends observed for non-atonic PPH (see Table S2). For PPH with blood transfusion, only atonic PPH with blood transfusion increased significantly (analyses by PPH subtypes and rates of blood transfusion by subtype are summarised in Tables S2 and S3).

Changes between 2001 and 2009 in maternal, fetal, and obstetric factors are summarised in Table 1. Risk factors for atonic PPH that increased over the study period included maternal age ≥35 years, primiparity, overweight and obese pre-pregnancy BMI, gestational age <28 weeks and 32–36 weeks, multiple pregnancy, and previous caesarean delivery. Obstetric factors that increased significantly were caesarean delivery, oxytocin augmentation, epidural analgesia, polyhydramnios, pre-eclampsia, prolonged second stage of labour, third- or fourth-degree perineal tears, combined forceps and vacuum deliveries, and chorioamnionitis.

**Table 1:** Temporal trends in maternal, fetal, and obstetric characteristics in British Columbia, Canada, from 2001 to 2009 (*n* = 371 193 deliveries)

	Rate per 100		2009 vs 2001		*P*
			
	2001	2009	RR	95% CI	
**Maternal age (years)**					
<20	4.40	3.23	0.73	0.68–0.79	<0.001
20–24	15.5	13.9	0.90	0.87–0.92	<0.001
25–29	28.5	28.5	1.00	0.99–1.02	0.84
30–34	31.9	32.0	1.01	0.99–1.02	0.005
35–39	16.5	18.3	1.11	1.08–1.15	<0.001
≥40	3.28	4.08	1.24	1.16–1.33	<0.001
**Parity**					
0	44.5	46.9	1.06	1.04–1.07	<0.001
1–2	49.9	47.9	0.96	0.95–0.97	<0.001
3–4	4.8	4.45	0.92	0.87–0.98	0.003
≥5	0.82	0.72	0.88	0.74–1.01	0.72
**BMI**					
Underweight (<18.5 kg/m^2^)	6.49	5.56	0.86	0.80–0.91	<0.001
Normal range (18.5–24)	62.7	60.3	0.96	0.94–0.97	<0.001
Overweight (25–29)	20.2	21.3	1.05	1.02–1.09	<0.001
Obese (≥30)	10.6	12.9	1.21	1.15–1.27	<0.001
**Birthweight (g)**					
<1500	1.11	1.28	1.15	1.01–1.31	0.02
1500–2499	3.57	4.01	1.12	1.04–1.20	0.002
2500–3999	80.5	82.2	1.02	1.01–1.02	<0.001
4000–4499	12.3	10.4	0.85	0.11–0.18	<0.001
≥4500	2.51	2.05	0.82	0.74–0.89	<0.001
**Gestational age (weeks)**					
<28	0.70	0.91	1.30	1.11–1.50	0.001
28–31	0.58	0.64	1.10	0.93–1.31	0.06
32–36	6.30	7.63	1.21	1.15–1.27	<0.001
37–41	90.7	89.5	0.99	0.98–0.99	<0.001
≥42	1.72	1.34	0.78	0.70–0.87	<0.001
**Current smoker**	12.3	8.86	0.72	0.69–0.75	<0.001
**Multiple pregnancy**	1.33	1.65	1.24	1.11–1.39	0.001
**Caesarean delivery**	26.7	30.2	1.13	1.11–1.16	<0.001
**Previous caesarean**	12.3	14.8	1.20	1.16–1.24	<0.001
**Epidural analgesia**	26.6	29.4	1.10	1.08–1.13	<0.001
**Induction**	21.2	20.0	0.94	0.92–0.97	<0.001
**Oxytocin augmentation**	14.8	16.1	1.09	1.05–1.12	<0.001
**Uterine rupture**	0.15	0.10	0.67	0.43–0.97	0.30
**Perineal tear (3rd or 4th degree)**	2.79	3.06	1.10	1.01–1.18	0.001
**High vaginal laceration**	0.39	0.20	0.51	0.39–0.66	0.001
**Cervical laceration**	0.21	0.20	0.95	0.69–1.25	0.97
**Placenta praevia**	0.63	0.68	1.08	0.90–1.26	0.18
**Placental abruption**	1.16	1.00	0.86	0.76–0.98	0.023
**Breech**	4.44	4.32	0.97	0.91–1.03	0.99
**Transverse lie**	0.39	0.33	0.85	0.67–1.05	<0.001
**Polyhydramnios**	0.58	0.77	1.33	1.11–1.55	<0.001
**Prolonged first stage**	4.33	3.53	0.82	0.76–0.87	<0.001
**Prolonged second stage**	7.88	8.35	1.06	1.01–1.11	0.004
**Pre-eclampsia**	1.07	1.20	1.12	0.99–1.28	<0.001
**Chorioamnionitis**	1.16	2.01	1.73	1.56–1.97	<0.001
**Forceps**	4.04	2.71	0.67	0.62–0.72	<0.001
**Vacuum**	7.17	7.13	0.99	0.94–1.04	0.28
**Forceps and vacuum**	0.37	0.63	1.70	1.40–2.08	<0.001

Rates expressed per 100 deliveries. Subjects with missing data were excluded from calculations.

Table 2 shows the crude and adjusted temporal increase in atonic PPH and the effects of various risk factors for atonic PPH. Known determinants of atonic PPH did not explain the temporal increase in atonic PPH: the crude 34% (95% CI 26–42%) increase in atonic PPH between 2001 and 2009 was not attenuated (42% increase, 95% 34–51%) after adjustment for changes in the listed determinants of PPH. Factors associated with atonic PPH are also shown in Table 2.

**Table 2:** Crude and adjusted temporal trends in atonic postpartum haemorrhage (PPH) and effects of maternal, fetal, and obstetric factors, British Columbia, Canada, 2001–2009 (*n* = 371 193 deliveries)

Determinant	Crude			Adjusted		
		
	OR	95% CI	*P*	OR*	95% CI	*P*
**Year**						
2002	0.99	0.93–1.06	0.88	1.01	0.95–1.08	0.71
2003	1.05	0.98–1.11	0.17	1.08	1.01–1.15	0.03
2004	1.00	0.94–1.07	0.95	1.05	0.98–1.12	0.18
2005	1.07	1.00–1.14	0.04	1.12	1.05–1.19	0.001
2006	1.10	1.04–1.17	<0.001	1.16	1.08–1.23	<0.001
2007	1.26	1.18–1.33	<0.001	1.34	1.26–1.43	<0.001
2008	1.39	1.31–1.48	<0.001	1.49	1.40–1.58	<0.001
2009	1.34	1.26–1.42	<0.001	1.42	1.34–1.51	<0.001
**Maternal age (years)**						
<20	1.11	1.02–1.20	0.01	1.04	0.96–1.13	0.30
25–29	0.94	0.90–0.98	0.01	0.95	0.91–1.00	0.04
30–34	0.94	0.90–0.98	0.01	1.00	0.95–1.04	0.84
35–39	0.91	0.87–0.96	<0.001	1.05	0.99–1.11	0.08
≥40	0.90	0.83–0.98	0.02	1.13	1.04–1.24	0.005
**Parity**						
0	1.51	1.47–1.56	<0.001	1.29	1.24–1.34	<0.001
3–4	1.00	0.93–1.08	0.98	0.95	0.88–1.02	0.16
≥5	1.03	0.86–1.24	0.73	0.94	0.78–1.12	0.48
**Birthweight (g)**						
<1500	0.45	0.37–0.55	<0.001	0.58	0.40–0.85	0.005
1500–2499	0.80	0.73–0.86	<0.001	0.71	0.64–0.78	<0.001
4000–4499	1.37	1.31–1.43	<0.001	1.49	1.43–1.56	<0.001
≥4500	1.59	1.46–1.73	<0.001	1.95	1.79–2.13	<0.001
**BMI (kg/m**^**2**^**)**	0.99	0.98–0.99	<0.001	1.00	0.99–1.00	0.04
**Gestational age (weeks)**						
<28	0.33	0.26–0.43	<0.001	0.46	0.29–0.73	0.001
28–31	0.77	0.64–0.94	0.01	1.16	0.88–1.53	0.28
32–36	0.84	0.79–0.89	<0.001	0.92	0.86–0.98	0.01
≥42	1.13	1.01–1.26	0.03	1.01	0.90–1.14	0.81
**Current smoker**	0.78	0.74–0.82	<0.001	0.83	0.79–0.88	<0.001
**Multiple pregnancy**	1.81	1.66–1.98	<0.001	3.55	3.20–3.94	<0.001
**Caesarean delivery**	0.31	0.30–0.33	<0.001	0.30	0.29–0.32	<0.001
**Previous caesarean**	0.43	0.40–0.45	<0.001	1.02	0.96–1.10	0.49
**Epidural analgesia**	1.54	1.50–1.59	<0.001	1.05	1.02–1.09	0.005
**Induction**	1.29	1.24–1.33	<0.001	1.16	1.12–1.20	<0.001
**Oxytocin augmentation**	1.49	1.44–1.54	<0.001	1.09	1.04–1.14	<0.001
**Uterine rupture**	1.27	0.87–1.85	0.21	2.59	1.67–4.02	<0.001
**Perineal tear (3rd or 4th degree)**	3.48	3.29–3.67	<0.001	1.82	1.71–1.93	<0.001
**High vaginal laceration**	6.07	5.28–6.97	<0.001	3.07	2.63–3.58	<0.001
**Cervical laceration**	10.40	8.90–12.16	<0.001	8.37	7.00–9.99	<0.001
**Placenta praevia**	1.34	1.15–1.56	<0.001	3.91	3.32–4.61	<0.001
**Placental abruption**	1.14	1.01–1.30	0.04	1.62	1.41–1.86	<0.001
**Breech**	0.32	0.29–0.36	<0.001	0.84	0.74–0.95	0.005
**Transverse lie**	0.66	0.49–0.89	0.01	1.41	1.03–1.93	0.03
**Polyhydramnios**	0.80	0.65–0.97	0.03	0.95	0.77–1.16	0.60
**Prolonged first stage**	1.73	1.63–1.84	<0.001	1.32	1.24–1.41	<0.001
**Prolonged second stage**	2.40	2.30–2.49	<0.001	1.39	1.32–1.46	<0.001
**Pre-eclampsia**	1.55	1.39–1.73	<0.001	1.81	1.61–2.04	<0.001
**Chorioamnionitis**	1.41	1.27–1.56	<0.001	1.63	1.46–1.82	<0.001
**Forceps**	3.68	3.50–3.86	<0.001	1.80	1.69–1.91	<0.001
**Vacuum**	1.88	1.79–1.96	<0.001	1.23	1.17–1.29	<0.001
**Forceps and vacuum**	3.38	2.99–3.81	<0.001	1.69	1.48–1.92	<0.001

Reference categories: calendar year 2001, age 20–24 years, parity 1–2, birthweight 2500–3999 g, gestational age 37–41 weeks, and absence of specified factor.

* Adjusted for all variables in the table.

Table 3 summarises the crude and adjusted temporal increase in atonic PPH in conjunction with blood transfusion ≥1 unit. Known determinants of atonic PPH did not explain the 51% (95% CI 11–104%) increase in atonic PPH with blood transfusion between 2001 and 2009 (adjusted increase 57%, 95% CI 15–115%). Similar results were observed for severe atonic PPH, as measured by the composite outcome of blood transfusion of ≥3 units or the use of procedures to control bleeding (Table 4): the crude 47% (95% CI 2–112%) increase in this composite outcome was not reduced upon adjustment for known determinants of severe atonic PPH (adjusted increase 47%, 95% CI 2–113%). Additional analyses carried out to assess temporal trends and adjustment for temporal trends among vaginal and caesarean deliveries showed the same patterns.

**Table 3:** Crude and adjusted temporal trends in atonic PPH and blood transfusion ≥1 unit and effects of maternal, fetal, and obstetric factors, in British Columbia, Canada, 2001–2009 (*n* = 371 193 deliveries)

Determinant	Crude			Adjusted		
		
	OR	95% CI	*P*	OR*	95% CI	*P*
**Year**						
2002	1.00	0.71–1.40	0.99	1.04	0.74–1.47	0.81
2003	1.19	0.86–1.66	0.28	1.24	0.89–1.73	0.20
2004	1.15	0.83–1.59	0.42	1.19	0.85–1.66	0.32
2005	1.40	1.02–1.92	0.04	1.43	1.04–1.98	0.03
2006	1.25	0.91–1.72	0.17	1.30	0.94–1.80	0.11
2007	1.37	1.00–1.87	0.05	1.44	1.05–1.98	0.03
2008	1.68	1.25–2.27	<0.001	1.77	1.30–2.40	<0.001
2009	1.51	1.11–2.04	0.01	1.57	1.15–2.15	0.004
**Maternal age (years)**						
<20	1.81	1.30–2.51	<0.001	1.75	1.25–2.45	0.001
25–29	0.86	0.68–1.09	0.21	0.82	0.65–1.04	0.10
30–34	1.01	0.81–1.25	0.95	0.93	0.74–1.16	0.52
35–39	1.03	0.80–1.31	0.83	0.94	0.73–1.22	0.66
≥40	1.59	1.13–2.23	0.01	1.29	0.90–1.85	0.17
**Parity**						
0	2.00	1.73–2.33	<0.001	1.30	1.06–1.60	0.01
3–4	1.28	0.88–1.84	0.19	1.29	0.88–1.90	0.19
≥5	2.33	1.24–4.37	0.01	2.53	1.33–4.80	0.005
**Birthweight (g)**						
<1500	1.40	0.79–2.48	0.25	0.84	0.23–3.03	0.79
1500–2499	1.64	1.21–2.21	<0.001	0.81	0.56–1.17	0.261
4000–4499	1.68	1.39–2.02	<0.001	1.78	1.46–2.16	<0.001
≥4500	2.00	1.39–2.89	<0.001	2.15	1.46–3.17	<0.001
**BMI (kg/m**^**2**^**)**	0.98	0.97–1.00	0.08	0.98	0.97–1.00	0.11
**Gestational age (weeks)**						
<28	0.98	0.44–2.21	0.97	1.06	0.23–4.83	0.94
28–31	1.87	0.97–3.60	0.06	1.37	0.46–4.01	0.57
32–36	1.67	1.34–2.08	<0.001	1.42	1.10–1.85	0.008
≥42	1.24	0.73–2.10	0.43	0.94	0.53–1.67	0.84
**Current smoker**	0.93	0.73–1.17	0.52	0.99	0.78–1.27	0.96
**Multiple pregnancy**	3.22	2.32–4.47	<0.001	2.79	1.93–4.06	<0.001
**Caesarean delivery**	1.34	1.16–1.55	<0.001	1.74	1.39–2.18	<0.001
**Previous caesarean**	0.85	0.69–1.05	0.14	0.95	0.70–1.29	0.75
**Epidural analgesia**	1.64	1.43–1.89	<0.001	0.90	0.74–1.08	0.25
**Induction**	1.42	1.22–1.67	<0.001	1.22	1.02–1.46	0.03
**Oxytocin augmentation**	1.36	1.14–1.62	<0.001	0.93	0.76–1.14	0.51
**Uterine rupture**	12.59	6.90–22.97	<0.001	8.45	3.90–18.32	<0.001
**Perineal tear (3rd or 4th degree)**	4.50	3.63–5.58	<0.001	2.75	2.13–3.55	<0.001
**High vaginal laceration**	18.66	13.45–25.89	<0.001	7.72	5.09–11.72	<0.001
**Cervical laceration**	41.15	30.75–55.06	<0.001	24.83	17.28–35.67	<0.001
**Placenta praevia**	6.55	4.61–9.30	<0.001	6.38	4.35–9.37	<0.001
**Placental abruption**	2.49	1.63–3.81	<0.001	1.81	1.14–2.88	0.01
**Breech**	0.86	0.60–1.24	0.42	0.75	0.51–1.11	0.15
**Transverse lie**	2.71	1.28–5.70	0.01	1.71	0.80–3.64	0.17
**Polyhydramnios**	1.82	0.94–3.51	0.08	1.34	0.68–2.62	0.40
**Prolonged first stage**	1.57	1.17–2.11	<0.001	1.25	0.91–1.71	0.17
**Prolonged second stage**	2.79	2.34–3.34	<0.001	1.46	1.17–1.82	0.001
**Pre-eclampsia**	3.97	2.84–5.56	<0.001	2.88	1.98–4.20	<0.001
**Chorioamnionitis**	3.29	2.37–4.57	<0.001	2.27	1.58–3.27	<0.001
**Forceps**	4.71	3.85–5.76	<0.001	3.32	2.49–4.41	<0.001
**Vacuum**	1.96	1.59–2.41	<0.001	2.22	1.73–2.86	<0.001
**Forceps and vacuum**	4.13	2.55–6.69	<0.001	3.11	1.79–5.39	<0.001

Reference categories: calendar year 2001, age 20–24 years, parity 1–2, birthweight 2500–3999 g, gestational age 37–41 weeks and absence of specified factor.

* Adjusted for all variables in the table.

**Table 4:** Crude and adjusted temporal trends in severe atonic PPH, defined using a composite outcome, * and effects of maternal, fetal, and obstetric factors, in British Columbia, Canada, 2001–2009 (*n* = 371 193 deliveries)

Determinant	Crude			Adjusted		
		
	OR	95% CI	*P*	OR**	95% CI	*P*
**Year**						
2002	0.83	0.54–1.27	0.39	0.86	0.56–1.32	0.49
2003	1.02	0.68–1.53	0.92	1.03	0.69–1.55	0.87
2004	1.48	1.02–2.14	0.04	1.49	1.03–2.17	0.04
2005	1.26	0.86–1.84	0.24	1.23	0.83–1.81	0.31
2006	1.26	0.86–1.85	0.23	1.27	0.86–1.86	0.22
2007	1.77	1.25–2.52	<0.001	1.80	1.26–2.58	0.001
2008	1.89	1.33–2.67	<0.001	1.89	1.33–2.69	<0.001
2009	1.47	1.02–2.12	0.04	1.47	1.02–2.13	0.04
**Maternal age (years)**						
<20	1.25	0.79–2.00	0.34	1.24	0.77–1.99	0.37
25–29	0.79	0.59–1.06	0.12	0.73	0.54–0.99	0.04
30–34	1.25	0.96–1.62	0.10	1.08	0.82–1.42	0.58
35–39	1.42	1.07–1.88	0.02	1.20	0.88–1.62	0.24
≥40	2.55	1.78–3.66	<0.001	1.87	1.28–2.74	0.001
**Parity**						
0	1.85	1.56–2.20	<0.001	1.39	1.09–1.77	0.008
3–4	1.44	0.97–2.13	0.07	1.44	0.96–2.18	0.08
≥5	1.20	0.45–3.23	0.71	1.24	0.45–3.37	0.68
**Birthweight (g)**						
<1500	1.38	0.71–2.67	0.34	1.08	0.22–5.35	0.93
1500–2499	1.77	1.27–2.47	<0.001	0.93	0.63–1.38	0.73
4000–4499	1.36	1.08–1.73	0.01	1.45	1.13–1.85	0.003
≥4500	1.75	1.12–2.74	0.01	1.85	1.16–2.95	0.01
**BMI (kg/m**^**2**^**)**	0.99	0.97–1.01	0.19	0.98	0.96–1.00	0.13
**Gestational age (weeks)**						
<28	0.89	0.33–2.39	0.82	0.66	0.09–5.07	0.69
28–31	1.96	0.93–4.14	0.08	0.88	0.25–3.09	0.84
32–36	1.73	1.35–2.22	<0.001	1.16	0.87–1.56	0.32
≥42	1.08	0.56–2.09	0.82	0.92	0.47–1.84	0.82
**Current smoker**	0.73	0.54–0.98	0.04	0.86	0.63–1.17	0.33
**Multiple pregnancy**	3.57	2.49–5.13	<0.001	2.80	1.85–4.23	<0.001
**Caesarean delivery**	2.01	1.71–2.37	<0.001	2.07	1.60–2.67	<0.001
**Previous caesarean**	1.08	0.86–1.35	0.52	0.97	0.69–1.36	0.86
**Epidural analgesia**	1.86	1.58–2.19	<0.001	1.27	1.03–1.57	0.03
**Induction**	1.11	0.92–1.35	0.28	0.95	0.76–1.18	0.62
**Oxytocin augmentation**	1.35	1.10–1.65	<0.001	0.87	0.68–1.10	0.25
**Uterine rupture**	22.04	12.86–37.77	<0.001	12.95	6.82–24.59	<0.001
**Perineal tear (3rd or 4th degree)**	3.46	2.62–4.56	<0.001	2.66	1.90–3.72	<0.001
**High vaginal laceration**	17.23	11.66–25.48	<0.001	7.61	4.65–12.44	<0.001
**Cervical laceration**	39.78	28.37–55.78	<0.001	25.12	16.69–37.81	<0.001
**Placenta praevia**	12.62	9.29–17.15	<0.001	9.89	7.00–13.97	<0.001
**Placental abruption**	3.74	2.48–5.63	<0.001	2.67	1.73–4.11	<0.001
**Breech**	1.02	0.69–1.51	0.94	0.73	0.48–1.12	0.15
**Transverse lie**	2.61	1.08–6.30	0.03	1.13	0.46–2.75	0.79
**Polyhydramnios**	3.04	1.67–5.52	<0.001	2.27	1.24–4.15	0.01
**Prolonged first stage**	1.46	1.03–2.07	0.04	1.13	0.78–1.65	0.52
**Prolonged second stage**	2.49	2.01–3.08	<0.001	1.50	1.15–1.96	0.003
**Pre-eclampsia**	2.64	1.65–4.22	<0.001	1.88	1.11–3.18	0.02
**Chorioamnionitis**	2.79	1.86–4.21	<0.001	1.65	1.05–2.60	0.03
**Forceps**	3.63	2.81–4.71	<0.001	2.25	1.55–3.26	<0.001
**Vacuum**	1.25	0.93–1.67	0.13	1.42	1.01–2.00	0.05
**Forceps and vacuum**	3.26	1.74–6.10	<0.001	2.15	1.06–4.35	0.03

Reference categories: calendar year 2001, age 20–24 years, parity 1–2, birthweight 2500–3999 g, gestational age 37–41 weeks, and absence of specified factor.

* Severe atonic PPH: blood transfusion ≥3 units or use of a procedure to control bleeding.

** Adjusted for all variables in the table.

Sensitivity analyses revealed that maternal pre-pregnancy factors (age, BMI, parity, smoking status, and previous caesarean delivery) and maternal pregnancy factors (multifetal gestation, pre-eclampsia, placenta praevia, placental abruption, chorioamnionitis, and polyhydramnios) attenuated the odds ratio for the rise in PPH with blood transfusion slightly (from an unadjusted OR of 1.50, 95% CI 1.11–2.04, to an adjusted OR of 1.45, 95% CI 1.06–1.96). The addition of the obstetric factors such as labour induction and labour augmentation, followed by epidural analgesia and caesarean delivery, made little difference to the odds ratio for the temporal increase in PPH with blood transfusion (Table S4). Results were similar for the other two outcomes (atonic PPH and the composite outcome). The study results did not differ when cases of missing BMI were included in the logistic regression analysis using multiple imputation techniques, as compared with complete case analysis.

## Discussion

Atonic PPH and severe atonic PPH increased in British Columbia, Canada, between 2001 and 2009. The rate of atonic PPH increased by 34% from 4.8% in 2001 to 6.3% in 2009, atonic PPH with blood transfusion ≥1 unit increased by 51% from 16.6 in 2001 to 25.5 per 10 000 deliveries in 2009, and atonic PPH with blood transfusion ≥3 units or use of procedures to control bleeding increased by 47% from 11.9 to 17.6 per 10 000 deliveries from 2001 to 2009. Logistic regression adjustment for the known determinants of PPH did not explain the aforementioned increase in atonic PPH.

It is unclear why atonic PPH and severe atonic PPH have increased, as the determinants examined accounted for little of the temporal increase. One possibility is that changes in diagnostic or coding practices were responsible for an artifactual increase in atonic PPH. Although there was no change in codes between ICD–9 and ICD–10 for PPH and its subtypes, a change in the chart abstraction methodology in Canadian hospitals with regard to PPH was implemented in 2006. The diagnosis of PPH, which was previously PPH as diagnosed and noted in the medical chart by the doctor, obstetrician, or midwife, was expanded to also include a documented blood loss in the medical chart of ≥500 ml for a vaginal delivery and >1000 ml for a caesarean delivery. The change in reporting may partly account for the increasing trend in atonic PPH among vaginal births after 2006, but cannot explain the steady pattern of increase in atonic PPH prior to 2006 among caesarean deliveries (Figure 1A), or the steady increase in severe PPH prior to 2006 as measured by several objective markers of severity (Figure 1B). Also, a general increase in the diagnosis of PPH would have led to similar increases in other subtypes of PPH such as PPH caused by retained placenta, secondary PPH, or PPH caused by coagulation defects (Table S2).

The increase in atonic PPH may have occurred because of changes in unmeasured risk factors or obstetric management. The Perinatal Data Registry began reporting cases of adherent placenta in 2009, and studies have reported increasing rates, especially in women with a previous caesarean delivery.^15^,^16^ However, given its rarity (16 per 10 000 deliveries in British Columbia in 2009), and as it would have been mostly classified as third-stage haemorrhage according to current coding standards, it is unlikely that changes in the frequency of adherent placenta underlie the increase in atonic PPH. The more liberal use of oxytocin during labour has been reported as a risk factor for PPH secondary to uterine atony (possibly caused by uterine muscle receptors becoming desensitised).^12^ We did not have information on the dose of oxytocin used for labour induction and/or augmentation. Additionally, we did not have information on use of magnesium sulfate, although we did control for pre-eclampsia. Magnesium sulfate has been associated with a four-fold increased risk of PPH among women with mild pre-eclampsia in one study, and hypotonic uterus may be a possible side effect.^17^,^18^ Furthermore, no information was available about the management of the third stage of labour, and it is unknown to what extent practice patterns changed over time, although increases in active rather than expectant management may be expected, especially in the most recent years since the first identification of increases in atonic PPH in Canada in 2007.^2^,^19^,^20^

Medications or environmental exposures during pregnancy may potentially underlie the increase in atonic PPH. Selective serotonin reuptake inhibitors (SSRIs) are commonly used in pregnancy (5% of pregnant women in British Columbia used these drugs in 2001),^21^ and have been reported to increase haemorrhage risk because of impaired platelet aggregation.^22^ Studies have shown a 30% (95% CI 0.98–1.72%) increased risk of PPH, as compared with a 12% (95% CI 0.62–2.01%) increased risk for non-SSRI antidepressant use.^23^ There also remains the disquieting possibility that a difficult-to-identify drug interaction underlies the increase in atonic PPH.

The increase in severe PPH may partly represent changes in practice patterns. In particular, there may be a more liberal use of blood transfusion at a lower threshold of PPH; however, blood transfusion in conjunction with other subtypes of PPH (as a result of retained placenta, secondary PPH, and PPH caused by coagulation defects) did not increase significantly (Tables S2 and S3). Similarly, the composite outcome in our study (that combined procedures to control bleeding along with blood transfusion ≥3 units, in order to partly account for potential changes in practice patterns) and several of the individual procedures to control PPH also showed substantial increases. Currently, the relative efficacy of the various procedures to control bleeding have not been adequately evaluated, although case series have reported these alternative strategies reduce rates of hysterectomy.^24^ This is confirmed by the small reduction in atonic PPH with hysterectomy observed in our study, when other procedures to control bleeding increased.

The literature on the relationship between BMI and PPH is conflicting, with some studies showing an increased risk of PPH among overweight and obese women,^8^ and other studies showing no excess risk.^17^ Our study indicated that increased BMI was not associated with an increased risk of atonic PPH or severe atonic PPH. More importantly, controlling for BMI did not explain the temporal increase in atonic PPH and severe atonic PPH. Our analysis on BMI was limited by missing data, although multiple imputation techniques for addressing this problem did not change the findings.

The strengths of our study include its population-based nature, the large study size, and the constancy of codes for PPH and its subtypes in ICD–9 and ICD–10. Our study was also able to examine the various surgical procedures used to control PPH. Reporting of blood transfusion is consistent in the database, as transfused women receive a specific form in their charts, so it is unlikely that reporting changes affected temporal trends. The limitations of our study include the retrospective nature of the study design and the potential for transcription and other errors in the database. Another minor limitation is that the definition of atonic PPH in ICD–9 and ICD–10 potentially includes PPH from causes other than atony (e.g. PPH resulting from a high vaginal laceration). Improvements in coding to isolate atonic PPH from other causes of PPH would aid future surveillance efforts.

In summary, our study confirms previous studies reporting increased rates of atonic PPH,^1^,^2^,^5^,^7^ and indicates that PPH, previously observed to have increased in the 1990s and early 2000s,^1^,^4^,^6^ has continued to increase in the late 2000s. Besides previously examined risk/protective factors for atonic PPH, we also controlled for pre-pregnancy BMI and other factors such as labour induction and oxytocin augmentation, but were unable to explain the temporal increase in atonic PPH and severe atonic PPH. Given the consistent findings across studies employing different definitions and markers of severity, improved PPH management strategies should be incorporated into routine clinical practice to reduce the severity of PPH and prevent long-term illness and disability.^24^ Future studies should continue to investigate novel risk factors for the increase in atonic PPH in order to identify and address causes.

### Disclosure of interests

The authors declare that they have no competing interests.

### Contribution to authorship

AM and KSJ were responsible for the intellectual content of the study proposal: they developed and articulated the conceptual framework and study design. AM, KSJ, and JAH developed the analytic approach. AM analysed the data and wrote the first draft of the article. JAH, LL, MSK, RML, and KSJ made substantial contributions to the study design, analysis, and interpretation of data, and revised the article for important intellectual content. All authors read and approved the final article.

### Details of ethics approval

This study received ethics approval from the University of British Columba Children & Women's Health Centre of British Columbia Research Ethics Board (project no. H11–00307).

### Funding

This work is supported by a Canadian Institutes of Health Research (CIHR) team grant in Severe Maternal Morbidity (MAH–115445). J.A.H. is the recipient of a CIHR New Investigator Award and a Scholar Award from the Michael Smith Foundation for Health Research. K.S.J. is supported by the Child and Family Research Institute.

## Supporting Information

**Table S1.** International Classification of Diseases (ICD–9 and ICD–10), the Canadian Classification of Diagnostic, Therapeutic and Surgical Procedures (CCP), and the Canadian Classification of Interventions (CCI) diagnosis/procedure codes used in the study.

**Table S2.** Temporal trends in atonic postpartum hemorrhage (PPH) and non-atonic PPH per 100 deliveries, and in PPH with blood transfusion (by subtype) per 10 000 deliveries in British Columbia, Canada, 2001–2009.

**Table S3.** Proportion of deliveries with any postpartum hemorrhage (PPH) subtype receiving a blood transfusion in British Columbia, Canada, 2001–2009.

**Table S4.** Odds ratio for temporal increase in atonic postpartum hemorrhage with blood transfusion (2009 versus 2001), adjusted for determinants of postpartum hemorrhage added incrementally to the model.
