# Supplementary material for: Epidemiological investigation of a temporal increase in atonic postpartum haemorrhage: a population-based retrospective cohort study
Source: BJOG. 2013 Mar 6;120(7):853–62. doi: 10.1111/1471-0528.12149 (PMC3717179; doi:10.1111/1471-0528.12149)
Supplement: Supplementary file 1 [file bjo0120-0853-SD1.pdf]

Table S1. International Classification of Diseases (ICD-9, ICD-10), the Canadian Classification of Diagnostic, Therapeutic and Surgical Procedures (CCP), and the Canadian Classification of Interventions (CCI) diagnosis/ procedure codes used in the study

Diagnosis/Procedure code	ICD-9	ICD-10	CCP	CCI
Postpartum hemorrhage	6660-6663	O720-O723		
- Due to retained placenta	6660	O720		
- Atonic postpartum hemorrhage	6661	O721		
- Secondary postpartum hemorrhage	6662	O722		
- Due to coagulation defects	6663	O723		
Hysterectomy			802;803	5MD60KE; 5MD60RC; 5MD60CB; 5MD60RD; 1RM87LAGX; (1RM89LA without 1PL74;1RS80; 1RS74)
Other procedures to control bleeding				5PC91LA, 1RM13, 1KT51;5PC91HT
-Suturing of uterus (e.g. b-lynch suture)				5PC91LA
-Control of bleeding using pelvic embolization				1RM13
-Ligation of pelvic vessels				1KT51
-Uterine (and vaginal) packing				5PC91HT
Uterine rupture	6650;6651	O710;O711		
High vaginal laceration	6654	O714		
Laceration of cervix	6653	O713		
Placenta previa	6410;6411;6421	O44		
Placental abruption	6412	O45		
Polyhydramnios	657	O40		
Prolonged first stage	6620	O630		
Prolonged second stage	6622	O631		
Preeclampsia	6424;6425	O14		
Chorioamnionitis	6584;7627	O411		

Table S2. Temporal trends in atonic postpartum hemorrhage (PPH) and non-atonic PPH per 100 deliveries, and in PPH with blood transfusion (by subtype) per 10,000 deliveries, British Columbia, Canada, 2001 – 2009.

	All years n	2001 n=39565	2002 n=39559	2003 n=39623	2004 n=39815	2005 n=40172	2006 n=41309	2007 n=43295	2008 n=43802	2009 n=44053	2001-2009 P for trend
**PPH (rates per 100 deliveries):**											
Atonic PPH	20144	4.8	4.8	5.0	4.8	5.1	5.3	6.0	6.6	6.3	<0.001
Non-atonic PPH*	5485	1.53	1.49	1.49	1.39	1.49	1.21	1.52	1.55	1.62	0.31
Due to retained placenta†	4328	1.19	1.16	1.18	1.12	1.17	0.97	1.14	1.26	1.28	0.25
Secondary PPH†	1168	0.34	0.32	0.33	0.29	0.32	0.23	0.37	0.29	0.35	0.84
Due to coagulation defects†	158	0.05	0.06	0.03	0.04	0.04	0.04	0.04	0.05	0.03	0.21
**PPH + blood transfusion (rates per 10,000 deliveries):**											
Atonic PPH + blood transfusion	800	16.7	16.7	19.9	19.1	23.4	20.8	22.9	28.1	25.2	<0.001
PPH due to retained placenta + blood transfusion	408	11.1	12.6	11.9	9.8	9.7	9.4	9.2	11.9	13.2	0.93
Secondary PPH + blood transfusion	132	2.8	3.3	3.3	2.8	4.5	1.7	4.6	4.3	4.5	0.09
PPH due to coagulation defects + blood transfusion	84	2.3	4.0	2.5	2.8	2.0	1.2	2.1	2.3	1.4	0.05
**Composite:** Atonic PPH + blood transfusion (>=3 units), hysterectomy, uterine suture, ligation or embolization, or uterine (and vaginal) packing	592	11.9	9.8	12.1	17.5	14.9	15.2	21.4	22.5	17.6	<0.001

*Excludes atonic PPH.

†May include more than one PPH subtype

Table S3. Proportion of deliveries with any postpartum hemorrhage (PPH) subtype receiving a blood transfusion, British Columbia, Canada, 2001-2009.

	All Years		% transfused			
PPH subtype	All years n	% transfused	2001	2002	2008	2009
Due to retained placenta	408	9.4	9.3	10.9	9.4	10.3
Atonic	800	4.0	3.5	3.5	4.3	4.0
Secondary	132	11.3	12.3	9.8	6.6	7.7
Due to coagulation defects	84	53.8	47.4	69.6	47.6	50.0

Table S4. Odds ratio for temporal increase in atonic postpartum hemorrhage with blood transfusion (2009 vs 2001) adjusted for determinants of postpartum hemorrhage added incrementally to the model

Adjusted for	Odds ratio	95% Confidence Interval
Crude odds ratio (2009 vs 2001)	1.508	1.112-2.044
Plus maternal pre-pregnancy factors: maternal age, BMI, parity, smoking status, previous cesarean delivery	1.479	1.090-2.006
Plus maternal pregnancy factors: multifetal gestation, preeclampsia, placenta previa, placental abruption, chorioamnionitis, polyhydramnios	1.445	1.064-1.963
Plus obstetric factors: labour induction and labour augmentation	1.451	1.069-1.971
Plus epidural analgesia	1.448	1.066-1.966
Plus cesarean delivery	1.448	1.066-1.966
